# Hypoglycemic Potential of *Carica papaya* in Liver Is Mediated through IRS-2/PI3K/SREBP-1c/GLUT2 Signaling in High-Fat-Diet-Induced Type-2 Diabetic Male Rats

**DOI:** 10.3390/toxics11030240

**Published:** 2023-03-01

**Authors:** Jeane Rebecca Roy, Coimbatore Sadagopan Janaki, Selvaraj Jayaraman, Vishnu Priya Veeraraghavan, Vijayalakshmi Periyasamy, Thotakura Balaji, Madhavan Vijayamalathi, Ponnusamy Bhuvaneswari, Panneerselvam Swetha

**Affiliations:** 1 Department of Anatomy, Bhaarath Medical College and Hospital, Bharath Institute of Higher Education and Research (BIHER), Chennai 600 073, Tamil Nadu, India; 2 Centre of Molecular Medicine and Diagnostics (COMManD), Department of Biochemistry, Saveetha Dental College & Hospitals, Saveetha Institute of Medical & Technical Sciences, Saveetha University, Chennai 600 077, Tamil Nadu, India; 3 Department of Biotechnology and Bioinformatics, Holy Cross College, Trichy 620 002, Tamil Nadu, India; 4 Department of Anatomy, Chettinad Hospital and Research Institute, Chettinad Academy of Research and Education, Chennai 603 103, Tamil Nadu, India; 5 Department of Physiology, Bhaarath Medical College and Hospital, Bharath Institute of Higher Education and Research (BIHER), Chennai 600 073, Tamil Nadu, India

**Keywords:** liver, T2DM, insulin resistance, *C. papaya*, in vivo, molecular mechanism, molecular docking

## Abstract

Regardless of socioeconomic or demographic background, the prevalence of type 2 diabetes mellitus, which affects more than half a billion people worldwide, has been steadily increasing over time. The health, emotional, sociological, and economic well-being of people would suffer if this number is not successfully handled. The liver is one of the key organs accountable for sustaining metabolic balance. Elevated levels of reactive oxygen species inhibit the recruitment and activation of IRS-1, IRS-2, and PI3K-Akt downstream signaling cascade. These signaling mechanisms reduce hepatic glucose absorption and glycogenesis while increasing hepatic glucose output and glycogenolysis. In our work, an analysis of the molecular mechanism of *Carica papaya* in mitigating hepatic insulin resistance in vivo and in silico was carried out. The gluconeogenic enzymes, glycolytic enzymes, hepatic glycogen tissue concentration, oxidative stress markers, enzymatic antioxidants, protein expression of IRS-2, PI3K, SREBP-1C, and GLUT-2 were evaluated in the liver tissues of high-fat-diet streptozotocin-induced type 2 diabetic rats using q-RT-PCR as well as immunohistochemistry and histopathology. Upon treatment, *C. papaya* restored the protein and gene expression in the liver. In the docking analysis, quercetin, kaempferol, caffeic acid, and p-coumaric acid present in the extract were found to have high binding affinities against IRS-2, PI3K, SREBP-1c, and GLUT-2, which may have contributed much to the antidiabetic property of *C. papaya*. Thus, *C. papaya* was capable of restoring the altered levels in the hepatic tissues of T2DM rats, reversing hepatic insulin resistance.

## 1. Introduction

The chronic metabolic condition, diabetes mellitus, is a major threat to society’s health and quality of life. Type 2 diabetes mellitus (T2DM), which affects more than half a billion people today, has been rapidly rising year after year, irrespective of socioeconomic or demographic status. If this number is not effectively managed, it will increase and have detrimental repercussions on people’s health, emotional, sociological, and financial status [1,2].

The liver is one of the primary organs in charge of maintaining metabolic homeostasis. Glucose homeostasis is maintained by coordinating the production of glucose in the liver through the pathways of glycogenolysis and gluconeogenesis in times of fasting, with the disposal of glucose into skeletal muscles through glycogen synthesis and glucose metabolism, and to a much lesser extent adipose tissue during feeding [3]. The functional duality of the liver in glucose production (glycogenolysis) and glucose storage (glycogenesis) helps in maintaining a fasting and fed state. This dynamic organ plays critical roles in many physiological processes, including the regulation of systemic glucose and lipid metabolism. Dysfunctional hepatic lipid metabolism is a cause of nonalcoholic fatty liver disease (NAFLD), the most common chronic liver disorder worldwide, and is closely associated with dyslipidemia, insulin resistance, and T2DM [4,5]. Hepatic insulin resistance occurs by means of excessive postprandial hyperglycemia due to inadequate inhibition of hepatic gluconeogenesis, decreased glycogen synthesis, and increased lipid accumulation [6,7,8]. Adipose tissue serves as the body’s energy reserve in times of nutritional excess by vigorously absorbing excessive blood glucose to store additional energy such as triglycerides. Moreover, insulin is essential for controlling the activity of lipolysis in adipose tissue, which is dysregulated in insulin resistance and results in the release of significantly elevated amounts of FFAs, pro-inflammatory cytokines (IL-1, IL-6, and TNF-), and glycerol into the bloodstream [8,9].

Kupffer cells, the localized macrophages of the liver, are involved in the production of cytokines and chemokines. They draw in new macrophages or other immune cells in response to foreign and local pro-inflammatory molecular triggers such as excess FFA and proinflammatory cytokines. Augmented levels of reactive oxygen species (ROS) reduce insulin receptor substrate -1 (IRS-1) and insulin receptor substrate -2 (IRS-2) recruitment and also the subsequent activation of the PI3K-AKT cascade downstream. Although they reduce hepatic glucose intake and glycogenesis, these signaling mechanisms also enhance hepatic glucose output, glycogenolysis, and triglyceride (TG) synthesis [10,11]. The pro-inflammatory cytokines produce acute-phase proteins in the liver, and may cause insulin resistance and apoptosis of pancreatic β cells [12,13]. Kupffer cells go from being anti-inflammatory to be pro-inflammatory, and it is assumed that this interaction with hepatocytes leads to insulin resistance [14]. This can also pave the way to the development of hepatic inflammation during non-alcoholic steatohepatitis (NASH) [15]. NAFLD is linked to more severe hyperinsulinemia, dyslipidemia, and insulin resistance in hepatic and adipose tissue in obese T2DM individuals than in those without NAFLD [16].

Modern medicine has grown in relation to efforts towards the development of antidiabetic medicine such as glycoside inhibitors as a way to reduce the absorption of carbohydrates so as to lower postprandial glucose and insulin levels [17,18].

In order to surpass the adverse effects of conventional diabetic treatments, seeking natural remedies is a definitive target. Our research focuses on finding therapeutic and preventive approaches that could slow the processes that lead to T2DM and enhance the treatment of issues related to diabetes. Thus, we intended to identify a natural plant-based product as a therapeutic approach to the effective management of diabetes mellitus. Several pieces of scientific literature have recorded the antidiabetic, immunomodulatory, and hepatoprotective outcomes of *Carica papaya* (*C. papaya*) [19,20,21,22]. In our previous work, we displayed the molecular action of the antihyperglycemic property of *C. papaya* in the skeletal muscle of T2DM animal models that reinstated glucose homeostasis via in vivo and in silico analysis. Our current study concentrates on the molecular mechanism of *C. papaya* in mitigating insulin resistance in the liver.

## 2. Materials and Methods

### 2.1. Chemicals

Eurofins Genomics India Pvt Ltd., Bangalore, India, and Sisco Research Laboratories, Mumbai, India, as well as other suppliers, provided all the chemicals, primers, reagents, and ELISA kits used in this work. The other suppliers were MP Biomedicals (Santa Ana, CA, USA); Sigma Aldrich (St.Louis, MO, USA); Spin React, Spain; Ray Biotech (Peachtree Corners, GA, USA); and Abbkine Scientific Co, Ltd. (Wuhan, China).

### 2.2. Collection of C. papaya Leaves

The leaves of *C. papaya* were gathered in Kerala and were desiccated in the shade, then pulverized. The National Institute of Siddha, Chennai, validated the content: -Certificate No: NISMB4392020.

### 2.3. Animals

At the Central Animal House of Saveetha Dental College and Hospital in Chennai, Tamil Nadu, male Wistar albino rats of 8–10 weeks old, weighing 150–180 g, were housed under standard environmental conditions of ambient temperature (21–2 °C), humidity (65–5%), and a stable 12-hour light–12-hour dark cycle. They were given regular rat pellets and unfettered use of water. (IAEC No: BRULAC/SDCH/SIMATS/IAEC/08-2021/071 dated 21 August 2021).

### 2.4. T2DM Induction

The rats were fed with high-fat diet (HFD) for 4 weeks, which included 66% conventional rat feed, 30% coconut oil, 3% cholesterol, and 1% cholic acid. Streptozotocin (STZ) (35 mg/kg) (Sigma Aldrich, St. Louis, MO, USA) was injected intraperitoneally to the rodents after 28 days of high-fat diet (HFD) feeding [23]. Two days after STZ administration, those animals with a fasting blood glucose of >120 mg/dl were taken into consideration for the study. Therefore, T2DM rats were allowed post induction.

### 2.5. Experimental Design

Random selection was used to choose five groups of eight rats each.

Group 1: Control rats
Group 2: T2DM-induced rats
Group 3: T2DM rats treated with ethanolic leaf extract of *C. papaya* (600 mg/kg bwt for 45 days)
Group 4: Metformin-treated T2DM rats (50 mg/kg bwt for 45 days)
Group 5: Control rats with ethanolic leaf extract of *C. papaya* (600 mg/kg bwt for 45 days).

In this study, we wanted to compare the efficacy of *C. papaya* with the commercially available oral hypoglycemic agent, metformin (50 mg/kg.b.wt). On the last day of the experiment, the animals were sedated with sodium thiopentone (40 mg/kg body weight) and blood was drawn through cardiac puncture. The blood was removed from the organs by injecting 20 mL of isotonic sodium chloride solution via the left ventricle. The liver from control and treated animals were immediately dissected and stored at −80 °C for further analysis.

### 2.6. Liver and Renal Function Markers

Urea and creatinine (kidney function markers), as well as liver function markers such as aspartate transaminase (AST) and alanine transaminase (ALT) were assessed using commercial kits.

### 2.7. Gluconeogenic Enzymes

#### 2.7.1. Assay for Glucose-6-Phosphatase

To assess glucose-6-phosphatase (G6P), Koide and Oda’s working protocol was used [24]. An hour was spent incubating 0.1 mL of the homogenized tissue with 0.3 mL of citrate buffer and 0.5 mL of substrate, and at 37 °C. 10 percent TCA was added to halt the reaction, and then Fiske and Subbarow’s method was used to calculate inorganic phosphate [25]. At 640 nm, the absorbance measurement was made.

#### 2.7.2. Assay for Fructose-1,6 Bisphosphatase

The Gancedo & Gancedo protocol was applied [26]. About 2.3 mL of a mixture with Tris-HCl buffer, potassium chloride, tissue homogenate, magnesium chloride, EDTA, and substrate was incubated for 15 min at 37 °C. The reaction was stopped using 10 percent TCA, and Fiske and Subbarow’s approach was used to estimate the endpoint [25].

### 2.8. Determination of Glycolytic Enzymes

The method described by Brandstrup et al. [27] was used to measure the activity of hexokinase (HK). HK converted ATP and D-glucose into glucose 6-phosphate and ADP, respectively. The residual glucose reacts with the o-toluidine reagent and emits a green color that can be observed at 640 nm spectomorphometrically. In terms of the mol of glucose phosphorylated per hour and mg of protein, the enzyme’s activity was calculated. Pyruvate kinase (PK) tissue activity was assessed by means of Valentine and Tanaka’s method [28]. Pyruvate generation from phosphoenolpyruvate was employed as a starting point. In order to determine how much pyruvate was released, dinitrophenyl hydrazine was supplemented, and the color formed was measured at 520 nm. The mol of pyruvate formed/min/mg protein was used to represent the values.

### 2.9. Glycogen Level

The Hassid and Abraham method [29] was used to estimate the amount of glycogen in the livers for all five study groups.

### 2.10. Oxidative Stress Markers

Rat liver tissues were examined using an ELISA kit for the detection of lipid hydrogen peroxide (H_2_O_2_) and peroxidation (LPO).

### 2.11. Enzymatic Antioxidants

Investigation of the expression of enzymatic antioxidants markers such as reduced glutathione (GSH), catalase (CAT), glutathione peroxidase (GPx), and superoxide dismutase (SOD) in the liver tissue of the rats were assessed with ELISA kit.

### 2.12. Total RNA, cDNA Synthesis, and Real-Time PCR

Total RNA was extracted from the liver of the rats in each of the five groups using the TRIR kit. The reverse transcriptase kit was provided by Eurogentec (Seraing, Belgium). The cDNA was created using 2 µg of total RNA. The list of primer sequences is mentioned in Table 1 as well as the house-keeping gene. The genes were amplified using a real-time PCR system (Stratagene MX 3000P, Poway, CA, USA) under the following reaction conditions: 40 cycles of 95 °C for 30 s, 59–60 °C for 30 s, and 72 °C for 30 s. Using the melt and amplification curves as a guide, relative quantification was created.

### 2.13. Histopathology

Hematoxylin and eosin were used to stain the liver tissue’s histopathology after it had been cut into sections and fixed in 10% neutral buffered formalin [34]. Sections were taken by means of microtome, and photographs at a 100-fold magnification were captured.

### 2.14. Immunohistochemical Analysis

Deparaffinized liver tissues from the experimental rats measuring 4 µm were then rehydrated using xylene and ethanol, sequentially, at steadily decreasing concentrations. The tissues were combined with sodium citrate buffer (1M, pH 6.0–6.2) and warmed for 5 min in three cycles. Thereafter, the slides were treated for 5 min with 1M PBS. Prior to processing the sections, the primary antibodies Akt and GLUT-2 were diluted 1:100 and peroxidase activity was performed.

### 2.15. Statistical Analysis

By means of Graph pad prism version 5 (computer-based software), the data were examined using one-way analysis of variance (ANOVA) and Duncan’s multiple range test to determine the importance of individual variance within the control and treated groups. The data were represented as mean ± S.E.M animals (n = 8) in a group and the significance was calculated at the levels of *p* < 0.05.

### 2.16. Molecular Docking

#### 2.16.1. Ligand Molecule Preparation

The literature on the bioactive substances found in *C. papaya* was compiled, and the PubChem database was used to download their chemical structures. Table 2 shows the list of ligands used in the study. In Pyrx, open Babel’s conjugate gradient technique was used to add hydrogens to the molecules while minimizing energy using the UFF force field. For pyrx 0.8 input, all structures were saved as pdb files. The Pdbqt file format was then used to save all of the ligand structures for later input into the AutoDock version. Later, the Auto Dock Pdbqt format was applied to all lead molecules.

#### 2.16.2. Protein Macromolecule Preparation

From the protein data bank, the three-dimensional crystal structures of IRS-2 (PDB ID: 3FQW) and P13K (PDB ID: 5XGJ) were downloaded. We eliminated all extra docking chains after downloading. The next step involved was the elimination of ligands and crystallized water molecules. Later, using a program developed at the Molecular Graphics Laboratory called the Mgl Tool (also known as Auto Dock tools), polar hydrogens and Kollmann charges were supplemented (MGL). They were added to the protein as a last step, along with any missing amino acids, and the protein as a whole was reduced using the Swiss PDB Viewer Software. The protein was subsequently set aside in the pdb format and was prepared for docking using Autodock Vina, with an estimation made using a soft virtual screening library by the name of Pyrx.

#### 2.16.3. Ligand–Protein Docking

Molecular docking experiments were conducted in order to comprehend the molecular interaction between the chosen drugs and the target proteins utilizing a computer technique. The binding mechanisms of the naturally occurring inhibitors from *C. papaya* were ascertained using the AutoDock (PyRx) suite of tools. The PyRx was used to assess the binding sites and the docking run of the target protein with the ligand. By selecting the Lamarckian GA docking technique and turning on the “Run AutoGrid” and “Run AutoDock” options in the control panel, an exhaustive search was carried out. This method involves the ligand randomly moving around the stationary protein. The grid point was given the energy of this one atom’s interaction with the protein. An equation based on free energy was used to determine interaction energies and include terms for dispersion/repulsion energy and directional hydrogen bonding.

## 3. Results

### 3.1. Efficacy of C. papaya on Liver and Renal Function Markers

**Figure 1:**
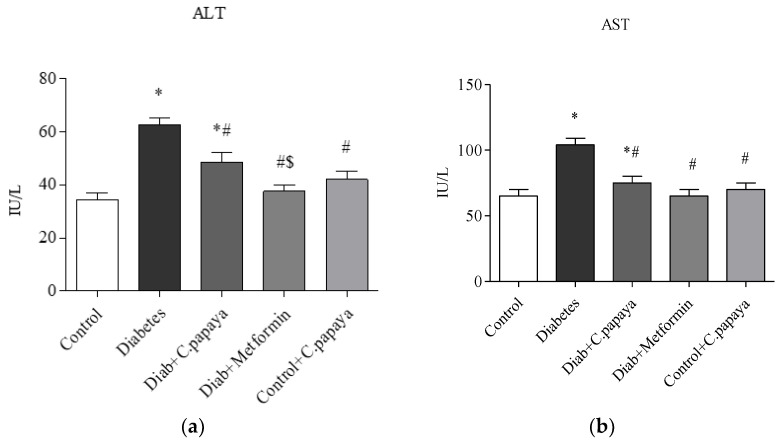
(**a**,**b**) Effect of *C. papaya* on (**a**) ALT and (**b**) AST levels. *p* < 0.05 was considered statistically significant among the groups: *-control; #-diabetes; $-*C. papaya*-treated T2DM.

**Figure 2:**
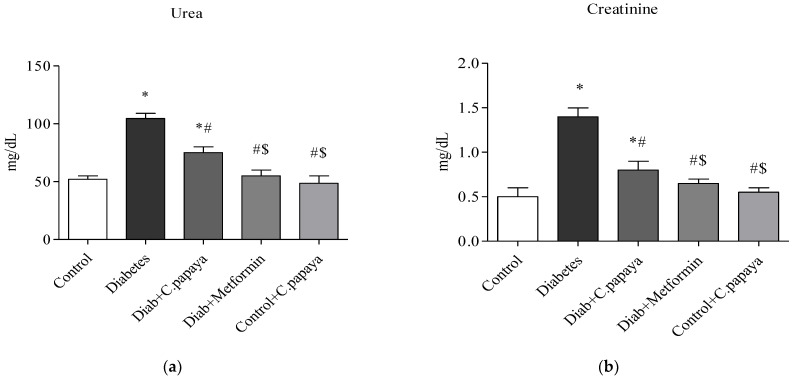
(**a**,**b**) Role of *C. papaya* on (**a**) urea and (**b**) creatinine levels. *p* < 0.05 was considered statistically significant among the groups: *-control; #-diabetes; $-*C. papaya*-treated T2DM.

The liver (ALT and AST) and kidney function markers (urea and creatinine) were significantly high (*p* < 0.05) in the T2DM Group 2 when matched with control rats and this is depicted in Figure 1 and Figure 2. The medicament with *C. papaya* reduced these high marker levels. The levels were brought down close to the control group with the administration of metformin. The control + *C. papaya* group showed nil changes.

### 3.2. Impact of C. papaya on Gluconeogenic Enzymes and Glycolytic Enzymes

**Figure 3:**
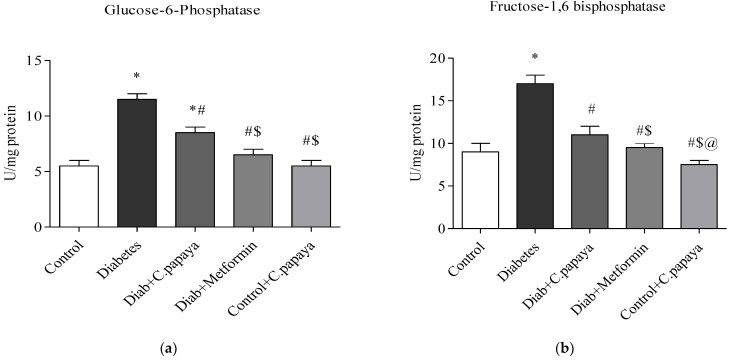
(**a**,**b**) Role of *C. papaya* on (**a**) G6P and (**b**) FBPase levels. *p* < 0.05 was considered statistically significant among the groups: *-control; #-diabetes; $-*C. papaya*-treated T2DM; @-T2DM rats + Metformin-treated T2DM.

**Figure 4:**
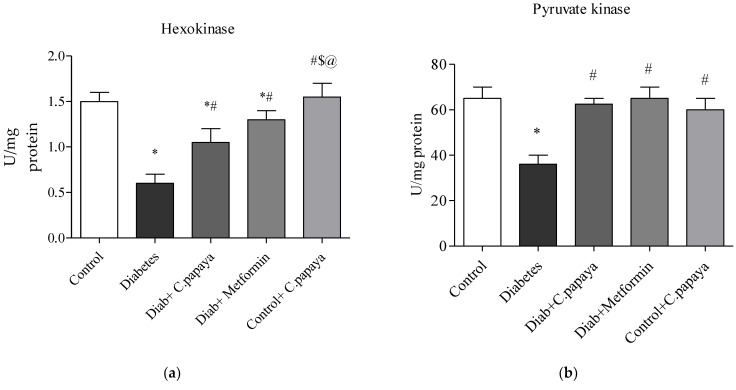
(**a**,**b**) Efficacy of *C. papaya* on (**a**) HK and (**b**) PK levels. *p* < 0.05 was considered statistically significant among the groups: *-control; #-diabetes; $-*C. papaya*-treated T2DM; @-T2DM rats + Metformin-treated T2DM.

Figure 3a,b demonstrates that in diabetic Group 2 rats, there is high fructose-1,6 bisphosphatase (FBPase) and glucose-6-phosphatase (G6P) activity. Treatment with *C. papaya* showed a reduction of these enzymes which was almost similar to the metformin group. Control + *C. papaya* did not exhibit any appreciable differences. The levels of hepatic hexokinase (HK) and pyruvate kinase (PK) in the rats of all five groups are showcased in Figure 4a,b. The levels were lowered in diabetic Group 2 when compared to Group 1. Furthermore, when *C. papaya* was administered orally, hepatic hexokinase and pyruvate kinase significantly increased (*p* < 0.05), in a similar way to the metformin medicament.

### 3.3. Outcome of C. papaya on Hepatic Glycogen Level

**Figure 5:**
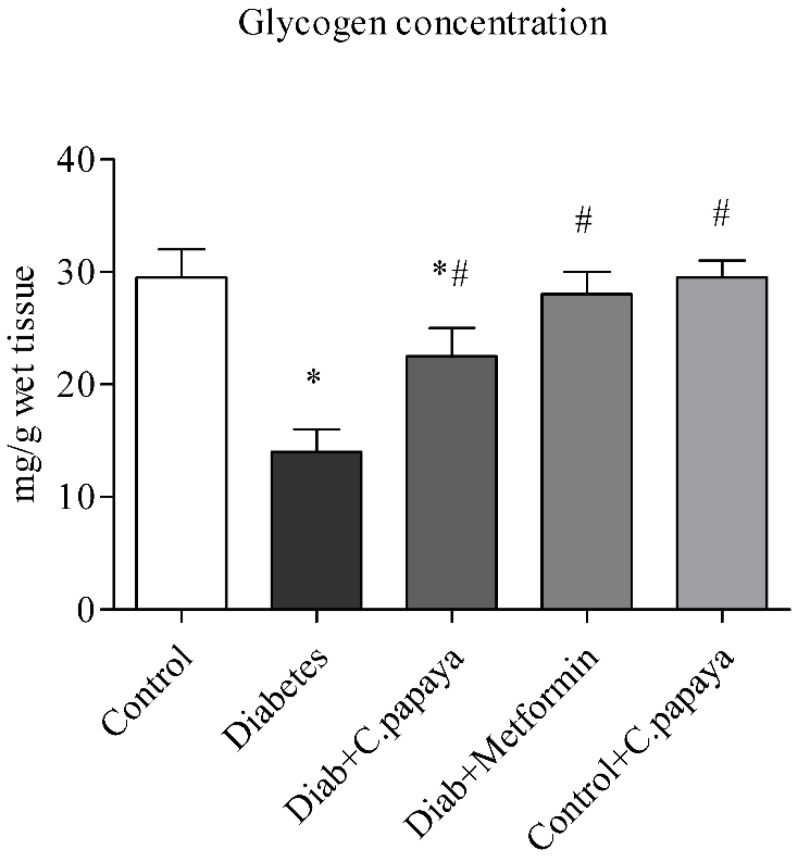
Role of *C. papaya* on hepatic glycogen concentration level. *p* < 0.05 was considered statistically significant among the groups: *-control; #-diabetes.

Figure 5 shows the levels of glycogen. Rats with T2DM in Group 2 have significantly lower (*p* < 0.05) levels of glycogen in their livers than rats without the disease in Group 1. This was largely restored by *C. papaya* therapy in Group 3, which is close to Group 4 with metformin therapy. Group 5 showed no alterations.

### 3.4. Efficacy of C. papaya on Oxidative Stress Markers

**Figure 6:**
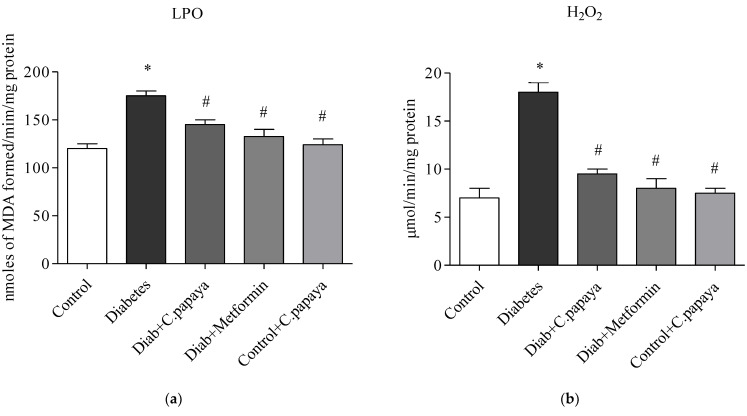
Role of *C. papaya* on (**a**) LPO and (**b**) H_2_O_2_ levels. *p* < 0.05 was considered statistically significant among the groups: *-control; #-diabetes.

Figure 6a,b illustrates the amounts of LPO and H_2_O_2_ in control and test rats. The levels of LPO and H_2_O_2_ in the liver of the T2DM group remained significantly greater (*p* < 0.05) when analyzed with Group 1. Indicators of stress effectively decreased values in the *C. papaya*-treated hepatic tissue when compared to Group 2. The levels were noticeably reduced by the drug metformin as well. In Group 5 rats, there were no changes in the levels of these markers of oxidative stress.

### 3.5. Impact of C. papaya on Enzymatic Antioxidants

**Figure 7:**
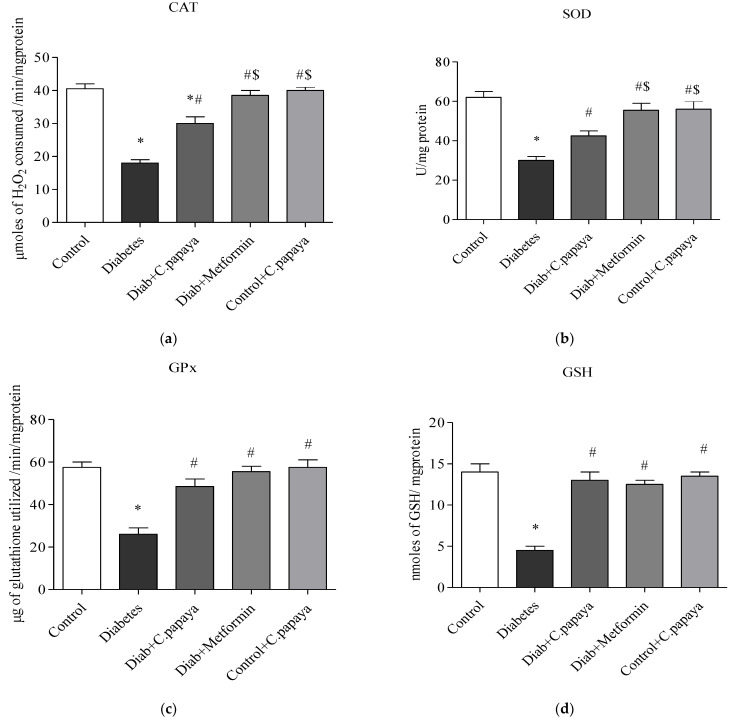
(**a**–**d**) Role of *C. papaya* on enzymatic antioxidants levels. *p* < 0.05 was considered statistically significant among the groups: *-control; #-diabetes; $-*C. papaya*-treated T2DM.

Figure 7a–d indicates the amounts of catalase, glutathione peroxidase, and superoxide dismutase in control and test rats. The enzymatic levels of GPx, CAT, GSH, and SOD were all significantly reduced (*p* < 0.05) in the liver of T2DM Group 2 when compared to Group 1. When compared to Group 2, the antioxidant enzyme values in the liver with the *C. papaya* treatment group were considerably improved. Additionally, their levels significantly improved when using the drug metformin. In Group 5 rats, these enzymatic antioxidant levels were constant.

### 3.6. Impact of C. papaya on mRNA Expression of IRS-2, PI3K, SREBP-1c and GLUT-2 in Liver

**Figure 8:**
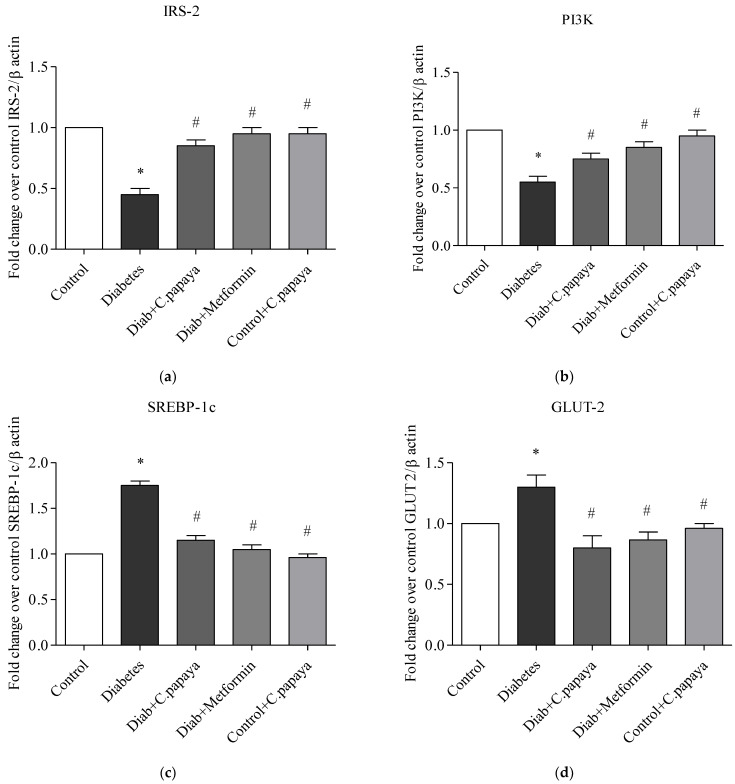
(**a**–**d**) Role of *C. papaya* on (**a**) IRS-2, (**b**) PI3K, (**c**) SREBP-1c, and (**d**) GLUT-2 mRNA expression levels. *p* < 0.05 was considered statistically significant among the groups: *-control; #-diabetes.

Figure 8a–d shows the impact of *C. papaya* on the mRNA expression of IRS-2, SREBP-1c, PI3K, and GLUT-2 in the liver of each of the five test groups. The mRNA gene levels of IRS-2 and PI3K remained significantly lowered (*p* < 0.05) in T2DM rats when compared with Group 1 rats. The administration of *C. papaya* enhanced these levels in the hepatic tissues in a similar way to metformin administration. Meanwhile, in diabetic rats, the levels of the mRNA genes for GLUT2 and SREBP-1c were dramatically elevated. Similar to the standard medication metformin, *C. papaya* therapy in diabetic rats decreased the levels of mRNA for GLUT2 and SREBP-1c in the liver. *C. papaya*-treated control rats did not display any discernible modifications.

### 3.7. Role of C. papaya on the Liver Tissue’s Histopathological Changes

**Figure 9:**
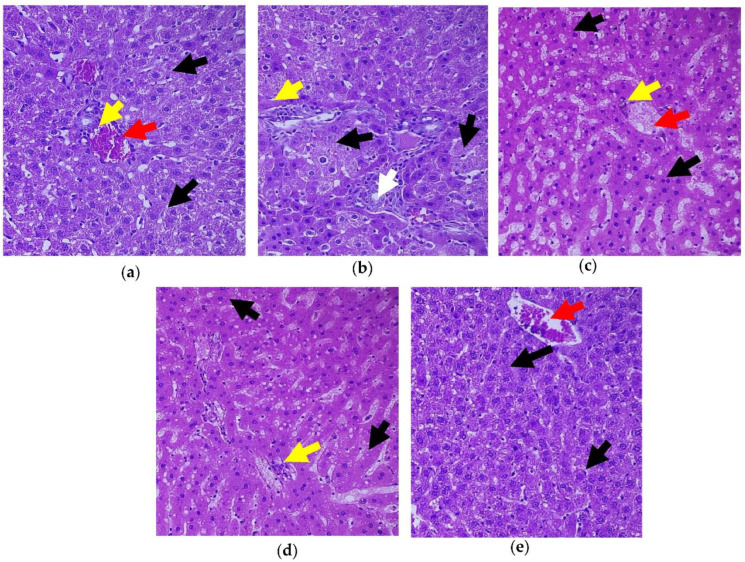
Histopathology of hepatic tissue displaying the effect of *C. papaya.* (**a**) Control rats; (**b**) T2DM rats presented degenerated hepatocytes (black arrow) while matched with the control; (**c**) *C. papaya*-treated T2DM rats manifested improved hepatocytes with hepatic bands; (**d**) metformin-treated T2DM rats exhibited the restored structure of hepatocytes; (**e**) *C. papaya*-treated control rats. Yellow arrow—centrilobular inflammation; white arrow—bile duct; red arrow—blood vessels.

To determine histological alterations in the diabetic condition and its restoration upon treatment with *C. papaya*, H&E staining was done (Figure 9a–e). The histology of the liver in the normal group revealed a single layer of hepatocytes encircling the central vein and a normal, distinct, and typical liver lobular architecture. However, the liver of Group 2 T2DM rats exhibited significantly extensive lipid vacuoles and a high number of fat depositions. The histological abnormalities and the micro-vesicular fatty alterations were seen to be significantly reduced by the *C. papaya* intervention in Group 3 in a similar way to that of the Group 4 metformin medicament, indicating that *C. papaya* could almost entirely restore the liver tissues to normalcy. No considerable changes were observed in group 5.

### 3.8. Efficacy of C. papaya on the Immunohistochemistry Alterations in Liver Tissue

**Figure 10:**
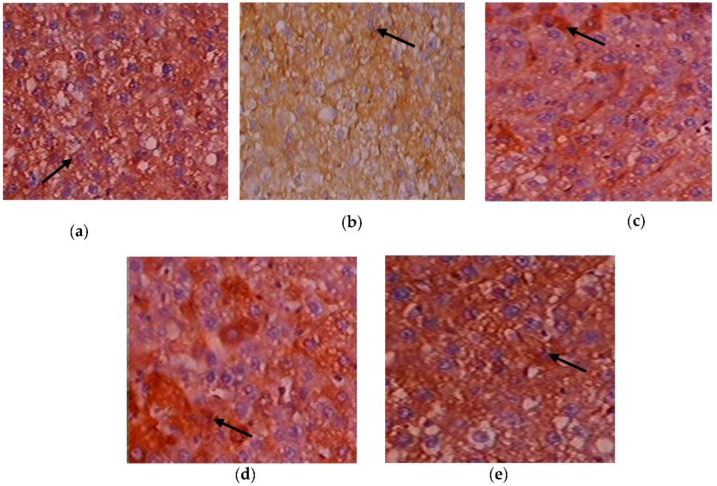
Expression of Akt via immunohistochemistry (100×). (**a**) Control rats; (**b**) T2DM rats; (**c**) *C. papaya*-treated T2DM rats; (**d**) metformin-treated T2DM rats; (**e**) *C. papaya*-treated control rats.

**Figure 11:**
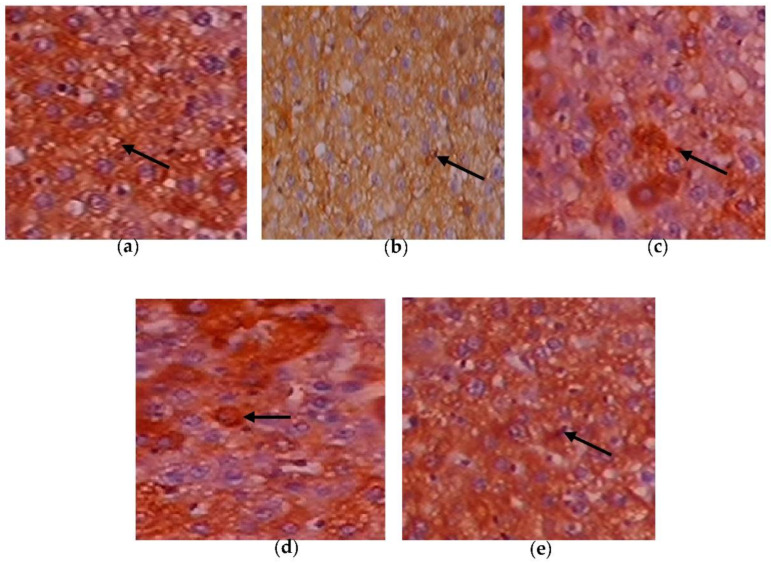
Expression of GLUT-2 via immunohistochemistry (100×). (**a**) Control rats; (**b**) T2DM rats; (**c**) *C. papaya*-treated T2DM rats; (**d**) metformin-treated T2DM rats; (**e**) *C. papaya*-treated control rats.

Figure 10a–e depicts the immunohistochemical changes in Akt in the study group. Akt expression in the liver was lower in Group 2 animals. As seen in Group 3, *C. papaya* therapy boosted the levels of Akt. The rats in Group 4 with metformin administration also displayed a considerable rise in Akt expression in the hepatic tissue of T2DM. Rats in Group 5 showed no discernible alterations. Figure 11a–e displays that the lessened expression of GLUT-2 in the liver of Group 2 T2DM rats were noticed when collated with Group 1 rats. The administration of *C. papaya* bettered the expression of GLUT-2 in Group 3 in a similar way to that of metformin therapy. No alterations were viewed in group 5.

### 3.9. Molecular Docking

**Figure 12:**
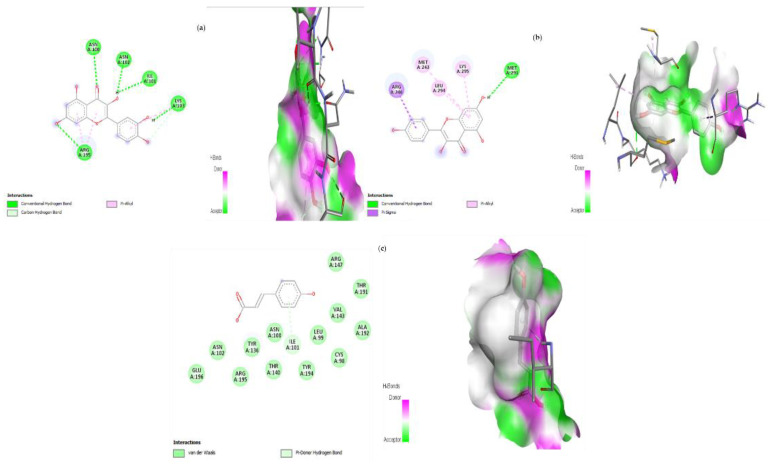
Lead compounds and their interaction with IRS-2 at the molecular level. (**a**) Quercetin; (**b**) kaempferol; (**c**) p-coumaric acid.

**Figure 13:**
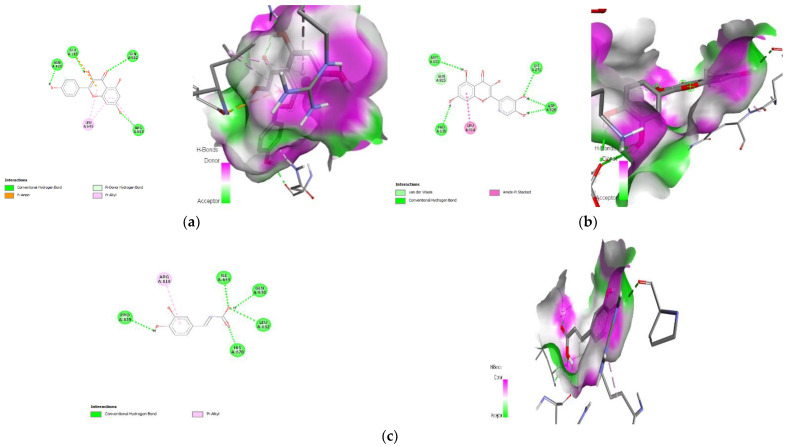
Lead compounds and their interaction with PI3K at the molecular level. (**a**) Kaempferol; (**b**) quercetin; (**c**) caffeic acid.

**Figure 14:**
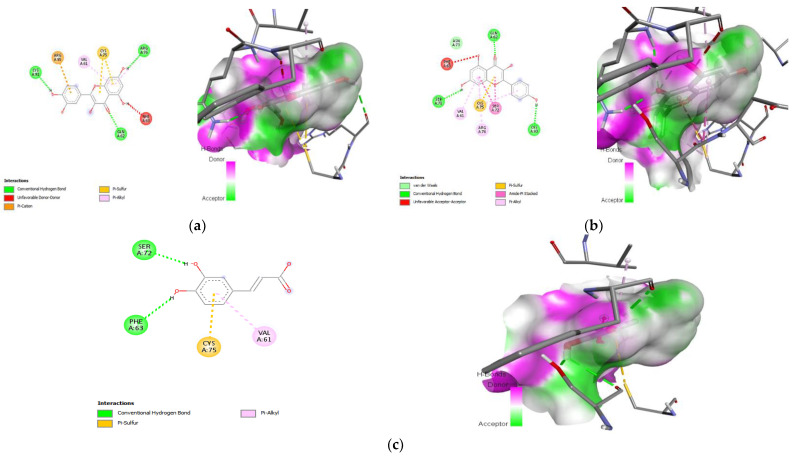
Lead compounds and their interaction with SREBP-1c at the molecular level. (**a**) Quercetin; (**b**) kaempferol; (**c**) caffeic acid.

**Figure 15:**
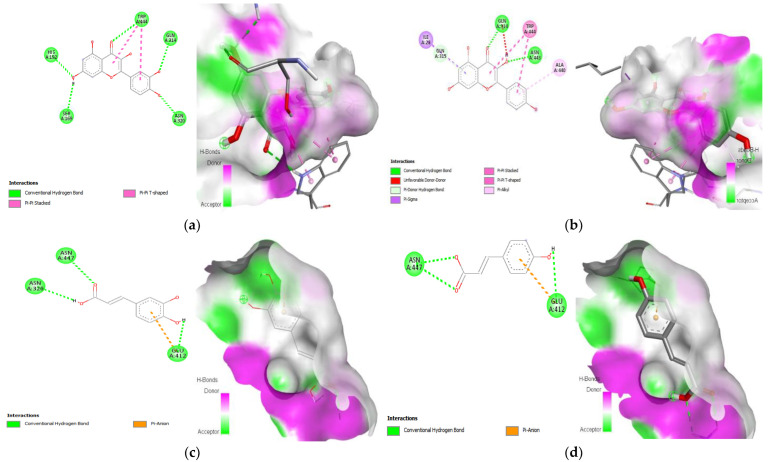
Lead compounds and their interaction with GLUT 2 at the molecular level. (**a**) Quercetin; (**b**) kaempferol; (**c**) caffeic acid; (**d**) p-coumaric acid.

Protein–ligand interaction is a result of molecular docking. The result of the protein–ligand interaction has been summarized in tabular form as the number of H-bonding and amino acid interactions, as well as the binding affinity score. The ligand’s negative binding energies signify a stable connection between the ligand and receptor. The binding energy and interaction of *C. papaya* compounds with the protein targets were made known in Table 3 and Figure 12, Figure 13, Figure 14 and Figure 15.

## 4. Discussion

The prevalence of NAFLD in obese people has increased exponentially, and it is considered the hepatic component of metabolic syndrome [35,36]. NAFLD usually appears before T2DM, and NAFLD individuals almost invariably have hepatic insulin resistance, which may play a key role in the pathophysiology from NAFLD to T2DM [37]. The liver serves as a significant site for the uptake and storage of glucose and can be responsible for up to one-third of oral glucose load disposal [38]. Increased hepatic glucose production is intertwined with fasting hyperglycemia in T2DM patients, suggesting that insulin resistance in the liver may play a role in hyperglycemia progression later on [39].

In the case of hepatic insulin signaling, insulin binds to and activates the insulin receptor tyrosine kinase (IRTK), which then facilitates the tyrosine kinase phosphorylation of insulin receptor substrates (IRS), most significantly IRS2 in the liver [40,41]. Proteins with Src homology 2 domains, such as phosphatidylinositol-3-OH kinase (PI3K), can bind to IRS2 by being phosphorylated [42,43]. Phosphatidylinositol-3,4,5-trisphosphate (PIP3) is recruited by PI3K binding to IRS2 in order to recruit Akt [43,44]. Akt activation serves four primary functions in the liver: (1) Increasing glycogen synthase (GS) activity to stimulate the synthesis of glycogen by inhibiting GS kinase (GSK); (2) Reduction of the expression of important gluconeogenic genes in part through forkhead box O1 (FoxO1) inactivation; (3) Sterol regulatory element-binding protein 1 (SREBP1) is controlled to stimulate endogenous fatty acid synthesis; and (4) Glucose transporter 2 (GLUT2) is directed to transport glucose into cells for aerobic metabolism or anaerobic breakdown [45,46,47,48,49].

Obesity causes ectopic fat accumulation in the liver and under the skin, which marks insulin resistance because adipocytes’ capacity to store and retain triglycerides is reduced [50]. Insulin-mediated inhibition of lipolysis in adipose tissue is weakened by insulin resistance, which leads to a significantly increased release of FFAs and glycerol into the bloodstream. Patients with NAFLD have higher amounts of circulating FFAs, which are the main source of escalating oxidative stress and inflammatory signals leading to systemic inflammation [50]. In hyperglycemic conditions, hepatic glucose production and glycogen synthesis is diminished while an increase in hepatic lipogenesis takes place [51,52]. Thus, hyperinsulinemia, hyperglycemia, and hypertriglyceridemia are common in T2DM patients [53].

Recent research has made significant efforts to completely understand the nature that bestows many medicinal benefits, and as a result, the quest for innovative approaches is conducted to effectively treat and defeat the growing epidemic of obesity and T2DM. As one of them, *C. papaya* is on the list of medicinal plants for the treatment of diabetes. *C. papaya* possess a rich source of phytochemicals with abundant anti-inflammatory and antioxidant properties, as well as favorable effects on glucoregulatory function [54]. In our earlier research, we found that *C. papaya* can restore glycemic control by exhibiting insulinemic action in T2DM skeletal muscle by increasing the levels of IR, IRS-1, Akt, and GLUT-4. The potential influence of *C. papaya* on insulin-signaling molecules in skeletal muscle was shown in a HFD-STZ-induced T2DM model [55,56]. In light of this, we centered on oxidative stress, gluconeogenic-glycolytic enzymes, and the expression of genes in the hepatic tissues of diabetic rats, and assessed the effect of *C. papaya* on hepatic insulin resistance using in vivo and in silico models.

Individuals with NAFLD and NASH generally have elevated circulating concentrations of markers of liver injury, such as AST, and ALT. Both AST and ALT serve as endoenzymes in hepatocytes for both amino acid production and catabolism and the alterations in the expression of ALT and AST in the serum serve as a biomarker for liver function [57]. In this work, the levels of liver function markers were increased in T2DM rats. The treatment with *C. papaya* leaf extract brought down the levels of ALT and AST in a comparable way to the result of metformin-treated rats, suggesting the hepatoprotective role of *C. papaya*. A parallel work by Abdel-Halim et al. [58] showed that the deleterious effect of carbon tetra chloride (CCl_4_) was eliminated upon the administration of *C. papaya*, which restored liver function markers. Albrahim and her co-worker displayed a considerable decline in ALT and AST levels in aged rats with the treatment of blueberry extract, thus proving its ability to enhance liver function [59]. NAFLD and type 2 diabetes may progress due to interactions between liver enzymes and insulin resistance. To establish the extent of renal impairment brought on by T2DM, serum urea and creatinine levels were also examined. According to earlier research, T2DM causes the kidney to expand due to hyperplasia and disrupts glomerular filtration [60]. Parallel to the study of Lathifi et al. [61], the renal function markers in our work were also lessened by enhancing the filtration function of the kidney upon the treatment of *C. papaya* in insulin resistance.

Due to hepatic insulin resistance in T2DM, the functionality of hepatocytes is dysregulated [62]. Thus, the levels of important enzymes involved in glycolysis and gluconeogenesis were quantified to investigate the molecular mechanism underlying the anti-hyperglycemia impact of *C. papaya* leaves. In the current study, enhanced hepatic glucose production and subsequent hyperglycemia in the HFD-streptozotocin-induced T2DM model were caused by the enhanced activity of FBPase and G6Pase, the major enzymes involved in gluconeogenesis in the liver. Further to this, the medicament of *C. papaya* downregulated the levels of these gluconeogenic enzymes close to those in the metformin-treated group. Similarly, the administration of *Scutellariae radix* and *Coptidis rhizome* reduced the levels of FBPase and G6Pase in a study carried out by Cui et al. [62]. Pari et al. [63] reported that when diabetic rats were given naringin in a dose-dependent manner, the altered FBPase and G6Pase activity were considerably restored to close to normal levels. Glycolysis typically has an impact on insulin output and various cell metabolisms. Deficiency of the key glycolysis enzymes, HK and PK, can result in decreased glycolysis, as well as decreased glucose absorption and utilization for energy output, which contributes to insulin resistance [64]. In the current work, poor insulin signaling resulted in a decrease in HK and PK in T2DM rats. In comparison to the metformin group, the medicament of *C. papaya* increased the range of HK and PK in the liver tissues of diabetic rats. According to Sureka et al. [65], another similar characteristic of *Sesbania grandiflora* augmented the levels of glycolytic enzymes in T2DM rats when matched with the control group. The total glycosides of *Cistanche tubulosa* were also used by Zhu et al. [66] to demonstrate higher levels of glycolytic enzymes in diabetic hepatic tissue. The results of our investigation suggested that the anti-hyperglycemic activities of *C. papaya* could be related to its phytochemical constituents which maintain glucose homeostasis [67].

Glycogen, which is mostly found in muscles and the liver, is crucial for preserving glucose homeostasis [68]. In a study by Luo et al., the amount of muscle and liver glycogen increased noticeably, showing that sweet potato leaf polyphenols may help diabetic mice produce more glycogen [68]. In a related, encouraging investigation, berry extract from *Aronia melanocarpa* increased the amount of hepatic glycogen in rats with type 2 diabetes [69]. Our treatment with *C. papaya* in T2DM rodents boosted the levels of hepatic glycogen in a similar way to that of the metformin administered group. The enhanced levels of hepatic tissue glycogen alleviated oxidative-stress-incited FFA and reactive oxygen species (ROS), thus improving the antioxidant defense status.

Increased ROS production from T2DM induced dyslipidemia and oxidative stress, which lead to lipid peroxidation and membrane damage [70]. In our investigation, the T2DM group was seen to have raised levels of LPO and H_2_O_2_, which increased the generation of ROS. In a related study, *Phyllanthus amarus* extract was shown to reduce oxidative stress biomarkers in HFD-induced T2DM rats [71]. However, *C. papaya* dramatically reduced LPO and H_2_O_2_ levels, owing to its antioxidant properties which come from its phytochemical backbone, which promotes the scavenging of overproduced ROS. Free radicals diminish enzymatic antioxidants such as CAT, SOD, GSH, and GPx in T2DM, thereby contributing to oxidative stress [72]. The treatment of *C. papaya* markedly boosted enzymatic antioxidant levels, which significantly decreased ROS and prevented lipid peroxidation in diabetic rodents’ liver tissue, and displayed a similar efficacy to the metformin-treated group. These results were pretty closely in accordance with Nain et al. study [73].

Earlier studies have recorded that the deterioration of glucose homeostasis due to IRS-2 and PI3K deficiencies led to insulin resistance in the liver [74,75]. This can result in the dysfunction of IRS-2 and PI3K which can contribute to the pathophysiology of T2DM [74]. In our current work, a decline in the levels of IRS-2 and PI3K was observed in T2DM rats. Zhang et al. [76] researched the antidiabetic effects of *Bifidobacterium animalis 01* and its beneficial improvement on IRS-1 and PI3K gene expression. Liu and his co-workers demonstrated that the derivatives of *Mogroside* delivered hypoglycemic results on HepG2 cells and lessened insulin resistance in T2DM rats by improving the gene expression related to insulin signaling [77]. In the same way, in our study, the therapy of *C. papaya* considerably improved the levels of IRS-2 and PI3K, in a way comparable to that of the metformin medicament. It is well known that in T2DM, liver GLUT2 gene transcription is elevated. This transcription factor plays a crucial role in the maintenance of glucose homeostasis [78]. The expression of the GLUT 2 gene is mainly dependent on sterol response element-binding protein (SREBP)-1c. SREBP-1c activates the GLUT2 promoter reporter, whereas a dominant-negative version of SREBP-1c inhibits the activation [79]. In the T2DM state, elevated ranges of SREBP-1c and GLUT-2 are observed. In our study, high levels of SREBP-1c and GLUT-2 were shown in the HFD-streptozotocin-induced T2DM rats. Similar works were reported in the earlier literature that reduced these levels to influence insulin signaling in the liver [80,81]. The medicament of *C. papaya* in our present research displayed the restoration of these upregulated genes in the insulin-signaling cascade, aiding in the maintenance of glucose homeostasis.

Insulin insufficiency and the dysregulation of fatty acid β-oxidation in mitochondria are the two main factors in the fatty degeneration of hepatocytes. This causes the conversion of fatty acids to numerous triglyceride droplets in the hepatocytes [82]. Kupffer cells get activated in inflammatory states such as obesity and T2DM, to release a good deal of inflammatory cytokines and chemokines [83]. As a result, hepatocyte injuries, cellular inflammation, fatty deposits, and vascular congestion were observed in the histopathological liver section of T2DM rats in our work, which was similar to Brancaccio et al. [84]. A comparable work was done by Motshakeri et al. [85]. The *C. papaya* treatment gradually reinstated the architecture of hepatocytes and consequently diminished cellular inflammation. These highlight the hepatoprotective potential of *C. papaya* and its role in hepatic insulin signaling.

**Figure 16:**
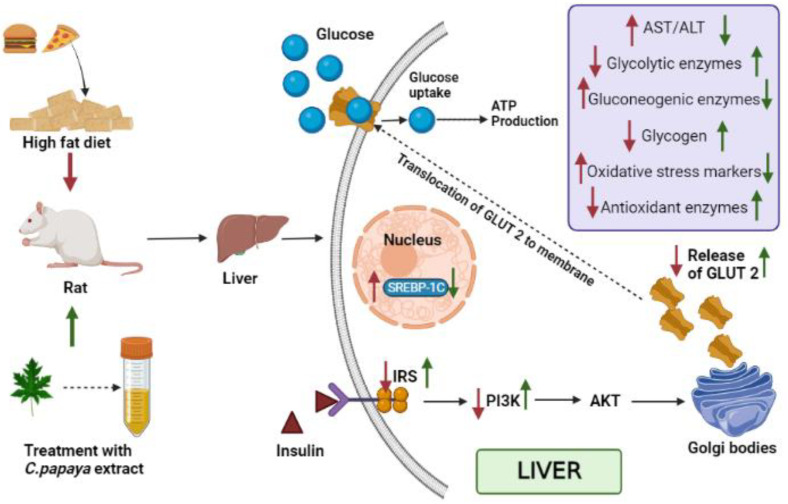
Summarizes the mechanisms of action of *C. papaya* leaf extract on IRS-2/PI3K/Akt/GLUT2. Signaling in hepatocytes. Red arrow indicates HFD-STZ-induced insulin resistance and metabolic dysfunction, while green arrow represents the therapeutic effects of *C. papaya* in the liver.

Hepatic insulin resistance can be alleviated by activating the PI3K/Akt pathway. In order to assess this, immunohistochemistry of Akt in hepatic tissues was done. The T2DM group displayed less expression of Akt in the liver tissues of the experimental rats. A similar study was done by Zhu et al. [86], in which the expression of Akt in T2DM-induced rodents was improved by the treatment of Liubao brick tea. In our work, the administration of *C. papaya* increased the levels of Akt in a comparable way to metformin treatment (Figure 16) The administration of *C. papaya* lowered the high levels in diabetic rats. A supportive work by Mathur and his team [87] found that *Psidium guajava Linn.* leaf extract reduced the levels of hepatic GLUT2. The excess availability of GLUT2 protein was decreased with the therapy of *C. papaya* to curb the glucose influx into hepatocytes. Thus, *C. papaya* improved downstream signaling by reducing gluconeogenic enzymes and oxidative stress markers, thus increasing glycolytic enzymes, hepatic glycogen content, and enzymatic antioxidants [88].

In the in silico analysis, non-covalent intermolecular interactions including hydrogen bonds between molecules, Van der Waal interactions, electrostatic interactions, and hydrophobic interactions all have an impact on binding affinity. The presence of several additional molecules may also affect the binding affinity of a ligand to the active site of a receptor. The outcomes showed that each of the ligands under investigation has a similar orientation and location within the putative binding site of the aforementioned proteins, which acts as a conduit intended for the substrate to reach the active site [89]. The strength of the relationship between the ligand’s affinity for the protein and the binding free energy can help interpret and comprehend the activity of the ligand through a variety of potential pathways. Additionally, the shape of the ligand–receptor complex plays a critical role in the development of pharmacological activity.

With regard to binding energy, hydrogen bond interaction, and hydrophobic interaction, quercetin, kaempferol, caffeic acid and p-coumaric acid demonstrated robust binding with every receptor in the current study. The researchers hunting for new drugs with anti-diabetic effects will benefit greatly from this in silico study. The possibility that the abovementioned proteins are intimately involved with *C. papaya’s* bioactive compounds needs to be confirmed in order to forward research.

## 5. Conclusions

The current study clearly demonstrates that *C. papaya* improves glycemic control in liver of HFD–STZ-induced T2DM rats through the regulation of IRS-2, PI3K, SREBP-1c, and GLUT-2 signaling molecules by facilitating glycolysis and inhibiting gluconeogenesis. This eventually encouraged the synthesis of hepatic glycogen by normalizing oxidative stress and antioxidant enzymes. In addition, the evidence from molecular docking analysis displayed that the compounds of *C. papaya* such as quercetin, kaempferol, caffeic acid and p-coumaric acid exhibited the best binding affinity against the targets IRS-2, SREBP-1c, PI3K, and GLUT-2. This study provides the foremost experimental evidence that *C. papaya* helps in maintaining glucose homeostasis in the liver against HFD-STZ-induced T2DM.

## Figures and Tables

**Table 1:** Details of primer sequences.

S.No	Gene Used	Primers’ Sequence	Ref
1.	Beta actin	Forward CCCGCGAGTACAACCTTCT Reverse CGTCATCCATGGCGAACT	[30]
2.	IRS-2	Forward CAAGAGTTCCAGCAGTAAC Reverse CAAGAGTTCCAGCAGTAAC	[31]
3.	PI3K	Forward CAAAGCCGAGAACCTATTGC Reverse GGTGGCAGTCTTGT TGATGA	[32]
4.	SREBP-1c	Forward GGAGCCATGGATTGCACATT Reverse AGGAAGGCTTCCAGAGAGGA	[30]
5.	GLUT-2	Forward GTCAGAAGACAAGATCACCGGA Reverse AGGTGCATTGATCACACCGA	[33]

**Table 2:** List of ligands.

Sl.No	Name of Compound
i.	Transferulic acid
ii.	Caffeic acid
iii.	Protocatechuic acid
iv.	Chlorogenic acid
v.	p-coumaric acid
vi.	Rutin
vii.	Quercetin
viii.	Kaempferol

**Table 3:** *C. papaya’s* top compounds and interactions and results with the target proteins.

S.No	Compound Name	Binding Energy Kcal/mol	Interacting Residues
IRS-2			
1.	Quercetin	−6	ASN-100 (H- bond) ASN-102 (H- bond) ILE-101 (H- bond) LYS-103 (H- bond) ARG-195 (H- bond)
2.	Kaempferol	−5.6	MET-291 (H-bond) ARG-246 (Pi-Sigma) MET-243 (Pi-Alkyl) LEU-294 (Pi-Alkyl) LYS-295 (Pi-Alkyl)
3.	p-coumaric acid	−4.8	ILE-101 (Pi donor H- bond) TYR-136 (Van der Waal)
PI3K			
1.	Kaempferol	−7.8	ARG-683 (H-bond) GLU-135 (H-bond) GLN-682(H-bond) ASN-428 (H-bond) LEU-645 (Pi-Alkyl)
2.	Quercetin	−7.7	MET-811 (H-bond) LYS-271 (H-bond) ASP-626 (H-bond) PRO-835 (H-bond)
3.	Caffeic acid	−6.6	ILE-633(H-bond) GLN-630 (H-bond) LEU-632 (H-bond) HIS-670 (H-bond) PRO-835 (H-bond) ARG-818 (Pi-Alkyl)
SREBP-1c			
1.	Quercetin	−7.7	CYS-93 (H-bond) GLN-62 (H-bond) ARG-76 (H-bond) VAL-61 (Pi-Alkyl)
2.	Kaempferol	−7.6	GLN-62 (H-bond) CYS-93 (H-bond) SER-78 (H-bond) ARG-76 (Pi-Alkyl) VAL-61 (Pi-Alkyl)
3.	Caffeic acid	−5.5	SER-72 (H-bond) PHE-63 (H-bond) VAL-61 (Pi-Alkyl)
GLUT-2			
1.	Quercetin	−8.9	HIS-192 (H-bond) SER-169 (H-bond) ASN-320 (H-bond) GLN-314 (H-bond) TRP-444 (H-bond)
2.	Kaempferol	−8.3	GLN-314 (H-bond) ASN-443 (H-bond) ALA-440 (Pi-Alkyl) ILE-28 (Pi- Sigma)
3.	Caffeic acidp-coumaric acid	−6.4	ASN-447 (H-bond) GLU-412 (H-bond) ASN-320 (H-bond) ASN-447 (H-bond) GLU-412 (H-bond)

## Data Availability

The data presented in this study are available in this article.
